# Collaborative Studies for the Detection of *Taenia* spp. Infections in Humans within CYSTINET, the European Network on Taeniosis/Cysticercosis

**DOI:** 10.3390/microorganisms9061173

**Published:** 2021-05-29

**Authors:** María Ángeles Gómez-Morales, Patrizio Pezzotti, Alessandra Ludovisi, Belgees Boufana, Pierre Dorny, Titia Kortbeek, Joachim Blocher, Veronika Schmidt, Marco Amati, Sarah Gabriël, Edoardo Pozio, Andrea Sylvia Winkler, The Ring Trial Participants

**Affiliations:** 1 Department of Infectious Diseases, Istituto Superiore di Sanità, Viale Regina Elena 299, 00161 Rome, Italy; patrizio.pezzotti@iss.it (P.P.); alessandra.ludovisi@iss.it (A.L.); or belgees.boufana@apha.gov.uk (B.B.); marco.amati@iss.it (M.A.); edoardo.pozio@iss.it (E.P.); 2 National Reference Laboratory for Trichinella & Echinococcus National Wildlife Management Centre (NWMC) Animal and Plant Health Agency Sand Hutton, York YO41 1LZ, UK; 3 Veterinary Helminthology Unit, Department of Biomedical Sciences, Institute of Tropical Medicine, 2000 Antwerpen, Belgium; pdorny@itg.be; 4 Center IDS, National Institute of Public Health and the Environment, RIVM, 3720 Bilthoven, The Netherlands; titia.kortbeek@rivm.nl; 5 Institute of Acute Neurology, Academic Teaching Hospital Feldkirch, Carinagasse 47, 6800 Feldkirch, Austria; joachim.blocher@gmail.com; 6 Center for Global Health, Department of Neurology, Technical University of Munich, 81675 Munich, Germany; veronika.schmidt@tum.de (V.S.); andrea.winkler@tum.de (A.S.W.); 7 Department of Veterinary Public Health and Food Safety, Faculty of Veterinary Medicine, Ghent University, 9820 Merelbeke, Belgium; Sarah.Gabriel@UGent.be; 8 Centre for Global Health, Institute of Health and Society, University of Oslo, 0318 Oslo, Norway

**Keywords:** cysticercosis, neurocysticercosis, *Taenia* spp., diagnosis, taeniosis, collaborative study, ring trial

## Abstract

Laboratory tools for diagnosing taeniosis/cysticercosis in non-endemic countries are available; however, there is little data on their performance. To provide information on the sensitivity, specificity, and reproducibility of these tools, inter-laboratory studies were organized within the EU COST-Action CYSTINET (TD1302). Two serological and one coprological Ring Trials (RTs) were organized to test a panel of human-derived sera and stool samples using assays routinely conducted by the participating laboratories to detect *Taenia* spp. infections. Four Western blots (WBs) and five ELISAs were used by nine laboratories for cysticercosis diagnosis. In the first serological RT, the overall sensitivity was 67.6% (95% CI, 59.1–75.4), whereas specificity was 97% (95% CI, 89.8–99.6). WBs recorded the best accuracy. A second serological RT was organized, to assess the three tests most frequently used during the first RT. Two out of six laboratories performed all the three tests. The overall sensitivity and specificity were 52.8% (95% CI, 42.8–62.7) and 98.1% (95% CI, 93.2–99.7), respectively. Laboratory performance strongly affected test results. Twelve laboratories participated in the coprological RT using conventional microscopy and six laboratories used molecular assays. Traditional diagnosis by microscopy yielded better results than molecular diagnosis. This may have been influenced by the lack of standardization of molecular tests across participating laboratories.

## 1. Introduction

Human cysticercosis (CC) is a zoonotic infection caused by the metacestode larval stage (cysticercus) of the pork tapeworm *Taenia solium*. In humans, cysticerci can establish in the central nervous system (neurocysticercosis, NCC), eye, muscle, sub-cutaneous, and, in rare cases, in other tissues. NCC is considered the most common helminth infection of the nervous system and is a major cause of epilepsy in low-income endemic countries [1,2]. Human taeniosis, caused by the presence of the adult tapeworm of *T. solium* or the beef tapeworm *Taenia saginata* in the gut, is not associated with major clinical symptoms but has significant implications as it allows the perpetuation of the parasite life cycle. Taeniosis caused by *T. solium*, represents a risk of NCC in tapeworm carriers and for people who live within the same environment. *T. saginata* causes human taeniosis and important economic losses in the bovine meat sector due to condemnation of carcasses of infected cattle, its natural intermediate host, harboring cysticerci [3,4]. In contrast to *T. solium*, *T. saginata* does not cause human NCC. A third human *Taenia* sp., *Taenia asiatica*, never recorded in Europe either as an autochthonous or as an imported case [5], was not included in the present study.

*Taenia solium* is highly endemic in Latin America, Asia, and sub-Saharan Africa where poor sanitation and free-ranging pigs with access to human feces contribute to the life cycle [6,7,8]. In the European Union member states and associated countries (henceforth EU), *T. solium* was endemic in the past, although recent publications suggest that autochthonous cases may still be acquired in some regions [9,10,11,12,13]. In recent years, imported NCC cases have increased in parallel to increased migration and travel [14]. In 2014, *T. solium* was ranked by an international panel of experts as the food-borne parasite of greatest global concern, affecting millions of individuals every year and causing substantial economic impact [15]. In 2016 and considering Europe as a whole, *T. solium* was ranked tenth among 27 parasites; however, when individual European regions were considered, this parasite had a higher ranking in eastern Europe [13,16].

Clinical manifestations of NCC are pleomorphic, variable, and non-specific, being related to differences in the number, size, location, and state of the parasite (calcified vs. viable cysts) and to the severity of the host’s immune response. Although no pathognomonic clinical picture exists, and in fact most patients remain asymptomatic for a long time, epileptic seizures and severe chronic progressive headaches are suggestive of NCC in endemic regions [17]. In non-endemic regions, the diagnosis of NCC is primarily based on neuroimaging, confirmed/aided by serology and a history of travel to or immigration from a *T. solium* endemic area [7,18,19]. The detection of taeniosis is most commonly made by microscopic examination of stool to detect eggs whose morphology and size are family specific (Taeniidae). Several in-house and commercial tools are used for the diagnosis of taeniosis/(neuro)cysticercosis by European clinical laboratories, [5]. However, data on their performance remains patchy. In order to address this issue, inter-laboratory collaborative studies (Ring Trials, RTs) were organized within the European Network on *Taeniosis/Cysticercosis* (CYSTINET) [20]. These studies aimed to determine the accuracy, sensitivity, specificity, and reproducibility of assays used by clinical laboratories in the detection of *Taenia* spp. infections in Europe. 

## 2. Materials and Methods

### 2.1. The European Network on Taeniosis/Cysticercosis: COST TD1302 Action CYSTINET

CYSTINET [20] is a multidisciplinary group of highly motivated scientists with substantial research output and expertise. It is recognized by the international community as a network of excellence. Between 2014 and 2017, CYSTINET members increased from 55 to over 150, representing 35 countries, and the group has remained active after the end of the COST grant period. The main objective of this Action was and still is to address the *T. solium/T. saginata* disease complexes from a One Health perspective through building a strong, extensive, multi-and interdisciplinary scientific network to induce sustainable collaborations and advance knowledge and understanding of taeniosis/cysticercosis. Specific objectives included the development of innovative and harmonized diagnostic and cost-efficient control tools, for which intra-European collaboration is essential to prevent the transmission of *T. saginata* and identify EU imported cases of taeniosis/cysticercosis caused by *T. solium*.

### 2.2. Collaborative Studies

During the COST TD1302 Action, CYSTINET members were invited via e-mail to express their interest in joining these collaborative studies. Participating laboratories are shown in Table 1. In the first RT, laboratories were invited to test a panel of human sera using their routine laboratory serological test/s, based on *T. solium* antibody or antigen detection. In the second RT, laboratories were invited to test a panel of human sera by the three assays that were determined by the first RT to be the most frequently utilized. In the third RT, laboratories were invited to test a panel of human stool by conventional microscopy (micro-coprological RT) and molecular (molecular-coprological RT) assays routinely used in their laboratories for the detection of *Taenia* infections (*T. saginata/T. solium*).

### 2.3. Panels of Samples

CYSTINET members provided serum and fecal samples to the European Union Reference Laboratory for Parasites (EURLP), Rome, Italy. These samples had been collected from confirmed patients following informed consent for their use in clinical studies and were stored under the required conditions (−20 °C or −80 °C) in respective laboratories for a number of years [21,22]. Upon arrival at the EURLP, samples were coded, and aliquots were sent out to the participating laboratories for blind analyses. Panels of samples for the two serological RTs are shown in Table 2.

A total of 12 serum samples, eight of which were from patients presenting clinical signs and symptoms suggestive of NCC, confirmed by cerebral computed tomography (CT) were used for the first serological RT. Of these 12, two were from healthy Italian individuals (used as negative reference samples), who, according to Italian law, are considered suitable for blood donation; one serum sample was from an Italian male with serological and ultrasound-confirmed cystic echinococcosis of the liver and one serum sample was from a 45-year-old Cuban female with cervical adenocarcinoma. Serum samples from NCC patients included in the first RT were categorized into two groups according to the localization of the cyst/s (intra-parenchymal or extra-parenchymal NCC) [23]. For the second serological RT, a panel of 16 samples was used, eight of which were from patients presenting clinical signs and symptoms suggestive of NCC confirmed by cerebral CT; however, data regarding cyst localization were not available for all samples. Six serum samples were from patients infected with other helminths, namely, *Trichinella spiralis* (*n* = 2), *Echinococcus granulosus* (*n* = 2), and *Opisthorchis felineus* (*n* = 2). Two serum samples were from Italian individuals considered suitable for blood donation. A total of five stool samples fixed in 95% ethanol were used for the third RT (microscopic and molecular-coprological RTs) (Table 3). The panel for microscopy testing, included two samples from healthy donors which served as negative controls, one sample from a healthy donor spiked with a known burden of *E. granulosus* eggs (1.5 × 10^3^ eggs/g of feces) and two samples from a confirmed *T. saginata* patient. One of these samples had a known *T. saginata* egg burden (10^3^ eggs/g of feces) and the second was spiked with egg-laden *T. saginata* verified proglottids. Samples 21, 22, and 23 from this panel should be classified positive for Taeniid eggs. In addition, for sample 23 the species identification could be reported (Table 3). The molecular-coprological RT panel included 3 samples from a healthy donor each spiked with 8 ng of *T. solium*, *T. saginata*, and *E. granulosus* verified DNA. A fourth sample was from a confirmed *T. solium* patient spiked with egg-laden *T. solium* verified proglottids. A fifth sample from a healthy donor completed the panel and served as a negative control. For this panel, samples 25, 28, and 29 should be classified positive (Table 3).

### 2.4. Instructions to Participating Laboratories 

Frozen serum samples stored in dry ice and fecal samples refrigerated using ice blocks, were dispatched to the participating laboratories by an international courier. Each laboratory received four forms as follows: 1. “Package check” to verify the content and the condition of the RT samples at arrival; 2. “Instruments and Materials” needed to perform the assays; 3.” Procedure” step by step description of the assay/s used by each laboratory; and 4. “Results” to record the result obtained for each sample. A confidential code was assigned to each laboratory, which was sent by email on the day of package shipment. 

### 2.5. Data Analysis

For serological and micro-coprological RTs, accuracy (or the degree of conformity of a measure to a true value; on the receiver-operator characteristic (ROC) curves, the accuracy is given by the area under the curve (AUC); sensitivity (the measure of how well a test can identify true positives); specificity (the measure of how well a test can identify true negatives); and inter-rater agreement (i.e., the degree of agreement among laboratories) were calculated using Stata v. 10 (StataCorp LLC, Lakeway Drive College Station, Texas, USA). Test agreement was expressed as kappa (K) index values. The scale of this measure of agreement ranges between ‘0’ when the level of agreement is what would be expected to be observed by chance and ‘1’, which indicates perfect agreement. For intermediate K index values, the following interpretations of agreement were used: 0.00–0.20: slight; 0.21–0.40: fair; 0.41–0.60: moderate; 0.61–0.80: substantial; and 0.81–1.00: almost perfect [24]. For molecular-coprological RT, diagnostic performance, i.e., sensitivity, specificity, and reliability, by test was calculated.

## 3. Results

### 3.1. First Serological RT

Ten laboratories participated in the first serological RT (Table 1). All laboratories but one received the panel of samples within 48 h of dispatch; the remaining laboratory received the samples four days after shipping. At delivery, the internal package temperature was in the range of +1.9 °C–−50 °C. The time between the arrival of the package at the laboratories and package control was less than 30 min. During this time, packages were stored at +4 °C. Samples were to be tested soon after the initial control check.

For antibody detection, 11 tests were performed by nine laboratories. One laboratory was unable to obtain the kit used in their routine testing and therefore did not participate. Some laboratories used more than one assay (Table 4).

The combined overall diagnostic performance of the tests used across the participating laboratories were as follows: sensitivity, 67.6% (95% CI, 59.1–75.4); specificity, 97% (95% CI, 89.8–99.6); accuracy, 77.4% (95% CI, 71.2–82.99). The results for sensitivity, specificity, reliability, and accuracy by test are shown in Table 5.

LLGP-EITB [26] and Cypress Diagnostics^®^ (Hulshoult, Belgium) showed the highest accuracy, i.e., 0.96 (95% CI 0.85–0.99) and 0.94 (95% CI 0.61–0.99), respectively. Considering both laboratory and test, the highest AUC (accuracy) was recorded for LLGP-EITB [26] performed by laboratory C (1.0, 95% CI 0.73–1.00; Table 6).

All tests but one were 100% specific, with only sample coded 11 testing falsely positive by NovaLisa ^®^ (Novatec Immundiagnostica GmbH, Dietzenbach, Germany) in laboratory C (Table 5 and Table 6). Focusing only on false negative results (18 samples), multilevel logistic analysis showed that the Odds Ratio (OR) for LLGP-EITB [26] vs. LDBIO Diagnostics^®^ (Lyon, France ) was 0.2 (*p* < 0.05, 95% CI 0.04–1.02), NovaLisa^®^ (Novatec Immundiagnostica GmbH, Dietzenbach, Germany) vs. LLGP-EITB [26] was 6.6 (*p* < 0.036, 95% CI 1.128–38.60), and NovaLisa^®^ (Novatec Immundiagnostica GmbH, Dietzenbach, Germany) vs. LDBIO Diagnostics^®^ (Lyon, France) was 1.32 (*p* < 0.66, 95% CI 0.375–4.645). The inter-rater agreement among laboratories performing the same test is shown in Table 7. 

The K index value for laboratories using LLGP-EITB [26] and LDBIO Diagnostics^®^ (Lyon, France) indicated a good level of agreement. A total of 39.2% and 11.8% of samples from individuals with intra-parenchymal and extra-parenchymal lesions, respectively, were classified as negative (false negative, Table 8).

Moreover, multilevel logistic analysis adjusted by laboratory (random effect) and adjusted by test (fixed effect) and laboratory (random effect), showed higher association for the intra-parenchymal localization of cysts with false negatives than the extra-parenchymal localization (Table 9).

For antigen detection, two tests were performed by three laboratories (Table 4). The highest accuracy was reported for an in-house ELISA used by laboratory E (DAET [28], AUC 0.81, 95% CI 0.51–0.99; Table 5). A similar accuracy was recorded by laboratory B (AUC 0.69, 95% CI 0.35–0.90) and laboratory C (AUC 0.62, 95% CI 0.35–0.90) using the commercial kit apDia^®^ (Turnhout, Belgium), with a K index value of 0.66 (95% CI 0.2–1.0; Table 6 and Table 7).

### 3.2. Second Serological RT

All six participating laboratories (Table 1) received the package within 24 h from dispatch and upon arrival all serum samples were still frozen. Only two laboratories tested the panel of serum samples by the three requested assays (LLGP-EITB [26], LDBIO Diagnostics^®^ (Lyon, France), and NovaLisa^®^ (Novatec Immundiagnostica GmbH, Dietzenbach, Germany); two laboratories tested the samples by LDBIO Diagnostics^®^ (Lyon, France) and NovaLisa^®^ Novatec Immundiagnostica GmbH, Dietzenbach, Germany); one by LLGP-EITB [26] and LDBIO Diagnostics^®^ (Lyon, France); and one by LDBIO Diagnostics^®^ (Lyon, France) (Table 4). Considering all the outputs of the six laboratories and the three tests together, sensitivity was 52.8% (95% CI 42.8–62.7), specificity 98.1% (95% CI 93.2–99.7), and accuracy 75.5% (95% CI 69.1–81.17). The most accurate test was LLGP-EITB [26] (AUC 0.8125, 95% CI 0.67–0.91; Table 5). Considering tests and laboratories, the highest AUC value was recorded for the LDBIO Diagnostics^®^ (Lyon, France) assay performed by laboratory C (AUC 0.94, 95% CI 0.69–0.99). One sample (sample 15) tested erroneously positive by two assays performed at the same laboratory (Table 6). When considering only false negatives, the multilevel logistic analysis showed that OR for LLGP-EITB [26] vs. LDBIO Diagnostics^®^ (Lyon, France) was 0.7 (*p* < 0.495, 95% CI 0.251–1.95) and NovaLisa^®^ (Novatec Immundiagnostica GmbH, Dietzenbach, Germany) vs. LLGP-EITB [26] was 3.82 (*p* < 0.02, 95% CI 1.24–11.69). The inter-rater agreement among laboratories performing the same test is shown in Table 7.

### 3.3. Third RT (Microscopic and Molecular-Coprological RTs)

Stool samples were sent refrigerated, and all participating laboratories received the package within 24 h. At arrival, the temperature inside the parcel was less than 15 °C. Twelve laboratories representing 11 countries participated in the microscopy RT (Table 1). Saline and iodine wet mount preparations of fecal smears for direct examination were carried out by seven laboratories, microscopic examination after concentration (whether by sedimentation or flotation) was performed in 11 laboratories, and Kato-Katz followed by Ziehl-Neelsen staining [29] was carried out in one laboratory (Table 10).

Eleven (91.7%) out of twelve laboratories correctly identified all the samples (Table S1). The highest accuracy was recorded for the direct examination of fecal smears after iodine and wet mount preparations (AUC 1.0, 95% CI 0.99–1.0, Table 11). All laboratories but one correctly identified Taeniid eggs in the three known positive microscopy RT samples. In contrast, *T. saginata* proglottids were recovered by only 4 (33%) laboratories. Of the 12 participating laboratories 91.7% correctly identified the negative controls, whereas a false positive was recorded by a single laboratory.

The six laboratories participating in the molecular-coprological RT (Table 1) used a number of different PCR protocols (Table 10). The performance of the assays used is shown in Table 12. No false positives were recorded by any of the participating laboratories for the negative control sample and also for the *E. granulosus* DNA-spiked sample (Table S2). Only 1 laboratory (17%) (Lab L) correctly identified all 3 positive samples and only a single laboratory (Lab S) registered false negatives for all the 3 true positive samples. Overall, 5 laboratories (83%) (B, C, D, E, L) detected *T. solium* spiked DNA-sample whereas only 1 laboratory (17%) (Lab L) correctly identified the *T. saginata* DNA-spiked sample. The sample spiked with *T. solium* proglottids was confirmed by 3 laboratories (50%) (C, D, L).

## 4. Discussion

Ring trials using well characterized clinical samples are essential to assess the performance of diagnostic tests, to harmonize and standardize their use, and importantly to ascertain their reproducibility and relevance to clinical work. The main limitations of this collaborative study include the low number of well characterized clinical samples; incomplete clinical information for some of the samples; and the lack of samples from patients with similar clinical presentations (such as brain tumours, metastases, or epileptic seizures) to establish specificity in clinical settings. The acquisition of clinical samples specific to regions where *Taenia* spp. infections have a rare occurrence as for instance in Europe is problematic [12,13,38]. However, despite these limitations, it has been possible to amass data on diagnostic tools used for the detection of *Taenia* spp. infections in humans as well as information on their performance in the hands of European laboratories. 

Laboratories participating in the first serological RT used 11 tests. Analysing all the results together, sensitivity was low (67.6%, 95% CI 59.1–75.4) and only 77.45% of the samples were correctly classified (i.e., correctely identified as positive samples). Four out of 11 tests were based on WB, five on ELISA and two were assays that are no longer commercially available (Table 4). In spite of studies showing the low diagnostic performance of these ELISAs [17,39,40,41], some European laboratories are still using these tests for NCC diagnosis [5] since these assays are easy to use particularly due to the convenience of automated reading. In recent years, several ELISA-based tests were developed and commercialized for the diagnosis of NCC. However, independent information regarding the specificity and sensitivity of these tests is often unavailable. The diagnostic specifications of these kits are reported in the kit insert, but usually they are not informative enough to establish the clinical performance of the assay. Since most of the laboratories have no or limited expertise on diagnosing NCC, the selection of a commercial kit for routine diagnosis can be difficult due to the lack of independent validation data, the lack of well-defined serum samples to validate the test and limited requests from clinicians. Additionally, shelf lifetime and cost are increasingly important, and diagnostic performance is difficult to establish. In the first serological RT, the LLGP-EITB [26] showed the best performance in terms of sensitivity (91.87%) and accuracy (0.958, 95% CI 0.85–0.99), followed by Cypress Diagnostics^®^ (Hulshoult, Belgium) (Table 5). The lowest accuracy was recorded for the CIE, which did not detect any positives and only correctly classified the negative samples, followed by Novagnost^®^ (0.6875 accuracy, 95% CI 0.35-0.90; Table 5). The in-house IFA, an outdated test which is no longer commercially available, showed similar or higher accuracy than some of the other tests such as Bioactiva Diagnostica^®^ (Bad Homburg, Germany), GTS-T24H [25], Novagnost^®^ (NovaTec Immunodiagnostica GmbH, Dietzenbach, Germany), NovaLisa^®^ (Novatec Immundiagnostica GmbH, Dietzenbach, Germany), r-EITB [27], r-T24H EITB [25], and LDBIO Diagnostics^®^ (Lyon, France ) (Table 5). However, the evaluation of a test used uniquely by one laboratory (i.e., Bioactiva Diagnostica^®^ (Bad Homburg, Germany), Cypress Diagnostics^®^ (Hulshoult, Belgium), GTS-T24H [25], Novagnost^®^,NovaTec Immunodiagnostica GmbH, Dietzenbach, Germany), r-EITB [27], r-T24H EITB [25], IFA, and CIE; Table 5) is negatively influenced by sample size, which may have led to the wide 95% CI observed in this study, when the accuracy was determined. In a previous study using a larger panel of samples [40], a sensitivity of 64.3% was recorded for Cypress Diagnostics^®^ (Hulshoult, Belgium), which is lower than the sensitivity (87.5%) detected in the present study, but similar to that of LLGP-EITB [26] (Table 5). In a recent study [41], the diagnostic value of r-T24H EITB [25] was considered similar to that of LLGP-EITB [26], whereas, in the present study, both tests performed differently (Table 5). This disparity may be related to the individual laboratories, since test accuracy may be influenced by laboratory performance. Additionally, the observed differences may be related to location, number and nature of cysts as well as to low IgG levels. The highest accuracy for the LLGP-EITB [26] test was achieved by laboratory C followed by laboratories B and L (Table 6). However, despite differences in accuracy, the K values show a good level of agreement among laboratories performing this test (Table 7). In the first serological RT, LLGP-EITB [26] was significantly less associated with false-negatives than LDBIO Diagnostics^®^ (Lyon, France) OR 0.2; 95% CI 0.04–1.02; *p* < 0.05) and NovaLisa^®^ (Novatec Immundiagnostica GmbH, Dietzenbach, Germany) (OR 6.6; 95% CI 1.128–38.60; *p* < 0.036). 

Most negative samples were correctly classified (i.e., correctely identified as negative samples). The influence of cyst localization in NCC patients was restricted to positive samples. Independent of the test and the laboratory, the percentage of false negative results was higher for samples from individuals with intra-parenchymal cysts (39.2%) than those from patients with extra-parenchymal lesions (11.8%) (Table 8). In addition, considering both laboratory or laboratory and test, a significantly higher OR (*p* < 0.004) was recorded for patients with intra-parenchymal lesions as opposed to those with extra-parenchymal ones (Table 9). This is in accordance with previous reports that described a higher antibody concentration in serum samples from extra-parenchymal NCC patients and, consequently, they are more easily detectable by a broad range of tests [17,23,42,43,44].

Only three laboratories tested the panel of sera by an antigen detection assay (Table 4). The known lower diagnostic sensitivity of this assay in comparison to those based on antibody detection [17,45] could be the reason for excluding this assay from the routine practice of many European laboratories. Sensitivities of 62.5% and 43.7% and accuracies of 0.81 and 0.66 were achieved for the commercial and in-house antigen detection assay, respectively (Table 5). In contrast to antibody detecting tests, antigen detecting assays only show positive results in the presence of viable cysticerci, which may explain their lower performance in this study. Antigen detection tests are of interest for the clinician to triage patients for neuroimaging in low-resource settings, support therapy options in association with the neuroimaging results and assess the outcome of anthelmintic treatment [17]. The inter-rater agreement for the circulating antigen test performed by two laboratories showed a K value of 0.66, which was considered substantial (Table 7). 

To confirm the results of the first serological RT, a second serological RT was organized. To this end, the three assays that were most frequently used during the first RT were selected, consequently increasing sample size per test. The total sensitivity was again low (52.8%). The LLGP-EITB [26] once more showed the highest performance (Table 5). These results support previous data showing higher diagnostic performance of WB in comparison to other assays [17,45]. Since laboratory performances are known to influence test results, laboratories should carefully check all the assay steps to ensure high standards are maintained. The participation of laboratories in consecutive RTs may positively affect laboratory performance. Furthermore, every new diagnostic assay should go through an external quality assessment service. 

The results of the copro RT suggest that generally the European laboratories appear to be better equipped for traditional microscopy than for molecular diagnosis of taeniosis, since only half of the laboratories participating in the microscopy testing also performed the molecular-coprological RT (Table 8). Using traditional coprological methods, 91.7% of laboratories correctly identified all the samples. This preference for the coprological tests, observed in a previous study [5], can be explained by the low cost associated with microscopic coprological diagnosis that is routinely performed for most intestinal parasites, including protozoa and helminths. The laboratory performances of the copro RT were very high with an accuracy close to 1 (Table 11). Conversely, the molecular methods did not reach the expected level of performance, showing a sensitivity lower than 75% (Table 11). 

Two DNA-spiked fecal samples were used in this copro RT due to difficulties in obtaining material from clinical patients. Despite this, spiked DNA of *T. solium* was retrieved by 83% of participants. In contrast only one laboratory was able to detect *T. saginata* spiked DNA. This may be a factor of both operator performance and sensitivity of the PCR assay used and is evident in the fact that only half of the participating laboratories were able to detect *T. solium* DNA in a sample from a confirmed patient with egg-laden proglottids (Table S2). Further to this, results recorded by the six participating laboratories were obtained using a mix of assays by which the positive RT samples were positive using a given PCR assay and negative with another (Table S2). This illustrates the need for the acquisition of clinical samples from confirmed taeniosis patients to be used in the standardization and harmonization of PCR protocols. This should be carried out within an external quality assessment service.

## 5. Conclusions

Based on the comparison of tests currently in use for the serological diagnosis of NCC by European laboratories, the use of a WB-based method appears advantageous. Laboratories should select the serological diagnostic kits based on their diagnostic performance rather than on cost and ease of use. For clinicians, our results confirm a high specificity, but relatively low sensitivity for *T. solium* antibodies or antigens.

To further evaluate the sensitivity and specificity of the assays tested in this study, additional biological samples positive for *Taenia* spp. from patients with confirmed taeniosis/cysticercosis originating from endemic regions should be tested.

The European laboratories performed well with regard to carrying out stool examination based on microscopy for detecting *Taenia* spp. infections. Copro-molecular techniques need standardization, validation and harmonization, and external quality assessment services. This is extremely important as molecular tools are relevant for *Taenia* species, allowing the identification of *T. solium* carriers, which may point the way to symptomatic or asymptomatic NCC cases and has clear therapeutic consequences. 

## Figures and Tables

**Table 1:** Laboratories and countries participating in the Ring Trials (RT).

Participating Laboratories	Country	1st RT	2nd RT	Micro-Copro RT	Molecular-Copro RT
Department of Medical Parasitology, Institute of Specific Prophylaxis and Tropical Medicine, Center of Pathophysiology, Infectiology and Immunology, Medical University of Vienna, Vienna	Austria	yes	yes	yes	yes
Institute of Tropical Medicine (ITM), Department of Biomedical Sciences, Veterinary Helminthology Unit, Antwerp	Belgium	yes	yes	yes	yes
Laboratory of Parasitology, Statens Serum Institut, Copenhagen	Denmark	no	no	yes	yes
Institute of Medical Microbiology, Immunology and Parasitology, University Clinic Bonn, Bonn	Germany	yes	no	no	no
Institute of Medical Microbiology, Immunology and Hygiene, Technical University of Munich	Germany	no	no	yes	no
Laboratory of Parasitology and Parasitic Diseases, School of Veterinary Medicine, Faculty of Health Sciences, Aristotle University, Thessaloniki	Greece	no	no	yes	no
Department of Infectious Diseases, Istituto Superiore di Sanità, Rome	Italy	yes	yes	yes	yes
Institute of Microbiology and Parasitology, Medical Faculty, University “Ss. Ciryl and Methodius”, Skopje	Republic of North Macedonia	no	no	yes	no
Center IDS, National Institute of Public Health and the Environment, RIVM, Bilthoven	Netherlands	yes	yes	yes	yes
Unidade de Parasitologia Médica, Instituto de Higiene e Medicina Tropical; Universidade NOVA de Lisboa	Portugal	yes	no	no	no
Mureic County Clinical Hospital, Mures	Romania	no	no	yes	no
Eco-Para-Diagnostic Medical Center, Bucharest	Romania	no	no	yes	no
Parasitological Laboratory at Clinic for Infectious and Tropical Diseases, Clinical Center of Serbia, Belgrade	Serbia	yes	no	no	no
Laboratory for Parasitology, Institute of Microbiology and Immunology, Faculty of Medicine, University of Ljubljana, Ljubljana	Slovenia	yes	yes	yes	no
Instituto de Salud Carlos III, Centro Nacional de Microbiología Parasitologia, Majadahonda, Madrid	Spain	yes	yes	yes	yes
SPDRL Glasgow Royal Infirmary, Glasgow	UK	yes	no	no	no

**Table 2:** Panels of human serum samples provided for the Ring Trials (RT).

	Sample Code	Sample Origin	Location of the Lesion
1st RT	1	Italian male with cystic echinococcosis	Liver
	2	Cape Verdean male with NCC	I ^1^
	3	Italian female travelling in South America with NCC	E ^2^
	4	Italian blood donor	NA ^3^
	5	Dutch female travelling in Asia with NCC	I
	6	Indian female with NCC	I
	7	Latin American male with NCC	E
	8	Mexican male with NCC	I
	9	Italian blood donor	NA
	10	Mexican male with NCC	I
	11	Cuban female with adenocarcinoma	NA
	12	Peruvian male with NCC	I
2nd RT	1	Italian male with trichinellosis	NA
	2	Italian male with cystic echinococcosis	Liver
	3	Italian male with opisthorchiasis	Biliary ducts
	4	Italian female travelling in South America with NCC	E
	5	Italian blood donor	NA
	6	Italian blood donor	NA
	7	Zambian male with NCC	U ^4^
	8	Zambian male with NCC	U
	9	Italian female living in Mexico with NCC	U
	10	Zambian male with NCC	U
	11	Zambian male with NCC	U
	12	South American male with NCC	U
	13	Italian male with trichinellosis	NA
	14	Italian male with cystic echinococcosis	NA
	15	Italian male with opisthorchiasis	Biliary ducts
	16	Italian male with NCC	U

^1^ intra-parenchymal; ^2^ extra-parenchymal; ^3^ not applicable; ^4^ unknown.

**Table 3:** Panel of stool samples provided for the coprological Ring Trials.

Test	Code	Sample Origin
Microscopic	20	Healthy donor
	21	*Taenia saginata* infected patient (10^3^ eggs/g)
	22	Healthy donor stool spiked with fixed *Echinococcus granulosus* eggs (1.5 × 10^3^ eggs/g)
	23	*T. saginata* infected patient (one mature proglottid)
	24	Healthy donor
Molecular	25	Healthy donor stool spiked with *Taenia solium* DNA (8 ng)
	26	Healthy donor
	27	Healthy donor stool spiked with *E. granulosus* DNA(8 ng)
	28	Healthy donor stool spiked with *T. saginata* DNA (8 ng)
	29	*T. solium* infected patient (one proglottid)

**Table 4:** Serological tests and number of tested samples performed by the laboratories in the course of the first and second Ring Trials (RT).

1st RT	Assays		Laboratories (Code)									
Test	Antibody Detection	A	B	C	D	E	F	G	H	J	L	Total
		No. of Tested Sera										
ELISA	*Taenia solium* IgG (Bioactiva Diagnostic^®^ _,_ Bad Homburg, Germany)	0	0	0	0	0	0	0	0	12	0	12
	Cysticercosis Antibody Kit (Cypress Diagnostics^®^, Hulshoult, Belgium)	0	0	0	0	0	0	12	0	0	0	12
	Enzyme-Linked ImmunoSorbent Assay (GST-T24H, in house) [25]	0	0	12	0	0	0	0	0	0	0	12
	Novagnost *Taenia solium* IgG (Novagnost^®^, NovaTec Immunodiagnostica GmbH, Dietzenbach, Germany)	12	0	0	0	0	0	0	0	0	0	12
	NovaLisa *Taenia solium* IgG ELISA (NovaLisa^®^, Novatec Immundiagnostica GmbH, Dietzenbach, Germany )	0	0	12	0	0	0	0	12	0	0	24
Western blot	Lentil lectin-bound glycoprotein enzyme-linked immunotransferblot assay (LLGP-EITB) [26]	0	12	12	0	0	0	0	0	0	12	36
	r-EITB [27]	0	12	0	0	0	0	0	0	0	0	12
	r-T24H EITB [25]	0	0	12	0	0	0	0	0	0	0	12
	CYSTICERCOSIS WB IgG (LDBIO Diagnostics^®^, Lyon, France)	0	12	0	12	0	12	0	12	0	0	48
Other	Counter immune-electrophoresis (CIE in house)	0	0	0	0	0	0	0	0	12	0	12
	Immunofluorescent Assay (IFA in house)	0	0	0	0	0	12	0	0	0	0	12
	Total	12	36	48	12	0	24	12	24	24	12	204
Test	Antigen detection											
ELISA	Antigen-ELISA kit (ApDia^®^, Turnhout, Belgium)	0	12	12	0	0	0	0	0	0	0	24
	Double antibodies ELISA (DAET in house) [28]	0	0	0	0	12	0	0	0	0	0	12
	Total	0	12	12	0	12	0	0	0	0	0	36
2nd RT												
Test	Antibody detection											
	Lentil lectin-bound glycoprotein enzyme-linked immunotransferblot assay (LLGP-EITB) [26]	0	16	16	0	0	0	0	0	0	16	48
	CYSTICERCOSIS WB IgG-(LDBIO Diagnostics^®^, Lyon, France)	0	16	16	16	16	0	0	16	0	16	96
	NovaLisa *Taenia solium* IgG-ELISA (NovaLisa^®^, Novatec Immundiagnostica GmbH, Dietzenbach, Germany)	0	0	16	0	16	0	0	16	0	16	64
	Total	0	32	48	16	32	0	0	32	0	48	208

**Table 5:** Diagnostic accuracy by test of the first and second Ring Trials (RT).

First RT Antibody Detection Test	TN ^1^	FP ^2^	FN ^3^	TP ^4^	Total Samples	Sensitivity (%)	Specificity (%)	Correctly Classified (Reliability) (%)	ROC Area (Accuracy)	95% CI
Bioactiva Diagnostica^®^ (Bad Homburg, Germany)	4	0	3	5	12	62.5	100	75.0	0.8125	0.51–0.97
Cypress Diagnostics^®^ (Hulshoult, Belgium)	4	0	1	7	12	87.5	100	91.7	0.9375	0.61–0.99
GTS-T24H [25]	4	0	2	6	12	75.0	100	83.3	0.8750	0.61–0.99
Novagnost^®^ (NovaTec Immunodiagnostica GmbH, Dietzenbach, Germany)	4	0	5	3	12	37.5	100	58.3	0.6875	0.35–0.90
NovaLisa^®^ (Novatec Immundiagnostica GmbH, Dietzenbach, Germany)	7	1	6	10	24	62.5	87.5	70.8	0.7500	0.53–0.90
LLGP-EITB [26]	12	0	2	22	36	91.7	100	94.4	0.9583	0.85.0.99
r-EITB [27]	4	0	2	6	12	75.0	100	83.3	0.8750	0.61–0.99
r-T24H EITB [25]	4	0	3	5	12	62.5	100	75.0	0.8125	0.51–0.97
LDBIO Diagnostics^®^ (Lyon, France)	16	0	10	22	48	68.8	100	79.2	0.8438	0.72–0.93
CIE	4	0	8	0	12	0	100	33.3	NE ^5^	NE
IFA	4	0	2	6	12	75.0	100	83.3	0.8750	0.61–0.99
Antigen detection test										
ApDia^®^ (Turnhout, Belgium)	7	1	9	7	24	43.7	87.5	58.3	0.66	0.45–0.84
DAET [28]	4	0	3	5	12	62.5	100	75.0	0.81	0.51–0.99
Second RT										
Antibody detection test										
LLGP-EITB [26]	23	1	8	16	48	66.67	95.83	81.25	0.8125	0.67–0.91
LDBIO Diagnostics^®^ (Lyon, France)	47	1	20	28	96	58.33	97.92	78.13	0.7812	0.65–0.86
NovaLisa^®^ (Novatec Immundiagnostica GmbH, Dietzenbach, Germany)	32	0	21	11	64	34.38	100	67.19	0.6719	0.54–0.78

^1^ True negative; ^2^ false positive; ^3^ false negative; ^4^ true positive; ^5^ not evaluable.

**Table 6:** Diagnostic accuracy by laboratory and test of the first and second Ring Trials (RT).

1st RT Antibody Detection Test	Lab Code	TN ^1^	FP ^2^	FN ^3^	TP ^4^	Total Samples	Sensitivity (%)	Specificity (%)	Correctly Classified (Reliability)(%)	ROC Area (Accuracy)	95% CI
LLGP-EITB [26]	B	4	0	1	7	12	87.5	100	91.7	0.9375	0.61–0.99
	C	4	0	0	8	12	100	100	100	1.0	0.73–1.0
	L	4	0	1	7	12	87.5	100	91.7	0.9375	0.61–0.99
LDBIO Diagnostics^®^ (Lyon, France)	B	4	0	3	5	12	62.5	100	75.0	0.8125	0.51–0.97
	D	4	0	2	6	12	75.0	100	83.3	0.875	0.61–0.99
	F	4	0	2	6	12	75.0	100	83.3	0.875	0.61–0.99
	H	4	0	3	5	12	62.5	100	75.0	0.8125	0.51–0.97
NovaLisa^®^ (Novatec Immundiagnostica GmbH, Dietzenbach, Germany)	C	3	1	3	5	12	62.5	75.0	66.7	0.6875	0.35–0.90
	H	4	0	3	5	12	62.5	100	75.0	0.8125	0.51–0.98
Antigen detection test											
apDia^®^ (Turnhout, Belgium)	B	4	0	5	3	12	35.0	100	58.3	0.69	0.35–0.90
	C	3	1	4	4	12	50.0	75.0	58.3	0.62	0.35–0.90
2nd RT											
Antibody detection test											
LLGP-EITB [26]	B	7	1	4	4	16	50.0	87.5	68.75	0.687	0.42–0.89
	C	8	0	2	6	16	75.0	100	87.5	0.875	0.62–0.98
	L	8	0	2	6	16	75.0	100	87.5	0.875	0.62–0.98
LDBIO Diagnostics^®^ (Lyon, France)	B	7	1	4	4	16	50.0	87.5	68.75	0.687	0.42–0.89
	C	8	0	1	7	16	87.5	100	93.75	0.937	0.69–0.99
	D	8	0	4	4	16	50.0	100	75.0	0.75	0.47–0.92
	E	8	0	4	4	16	50.0	100	75.0	0.75	0.47–0.92
	H	8	0	4	4	16	50.0	100	75.0	0.75	0.47–0.92
	L	8	0	3	5	16	62.5	100	81.25	0.812	0.54–0.95
NovaLisa^®^ (Novatec Immundiagnostica GmbH, Dietzenbach, Germany)	C	8	0	5	3	16	37.5	100	68.75	0.687	0.41–0.89
	E	8	0	6	2	16	25.0	100	62.5	0.625	0.35–0.85
	H	8	0	5	3	16	37.5	100	68.75	0.687	0.41–0.89
	L	8	0	5	3	16	37.5	100	68.75	0.687	0.41–0.89

^1^ True negative; ^2^ false positive; ^3^ false negative; ^4^ true positive.

**Table 7:** Inter-rater agreement of serological Ring Trials (RT).

1st RT Antibody Detection Test (Laboratory Code)	K	95% CI	*p*
LLGP-EITB [26] (B, C, L)	0.88	0.72–1.00	<0.001
LDBIO Diagnostics^®^ (Lyon, France )(B, D, F, H)	0.89	0.88–1.00	<0.001
NovaLisa^®^ (Novatec Immundiagnostica GmbH, Dietzenbach, Germany) (C, H)	0.50	0.02–0.98	0.04
Antigen detection test			
apDia^®^ (Turnhout, Belgium ) (B, C)	0.66	0.20–1.00	-
2nd RT Antibody detection test			
LLGP-EITB [26] (B, C, L)	0.36		0.006
LDBIO Diagnostics^®^ (Lyon, France) (B, C, D, E, H, L)	0.55		0.000
NovaLisa^®^ (Novatec Immundiagnostica GmbH, Dietzenbach, Germany) (C, E, H, L)	0.89		0.000

**Table 8:** Influence of the cyst localization on the results of the first serological Ring Trial.

False Negative	Intra-Parenchymal	Extra-Parenchymal	Total
	N (%)	N (%)	
No	62	30	92
Yes	40 (39.2)	4 (11.8)	44
Total	102	34	136

**Table 9:** Odds Ratio (OR) of the intra-parenchymal versus extra-parenchymal cysts on the serological results of the first Ring Trial.

False Negative	OR	95% CI (*p*-Value)	Note
Intra-parenchymal vs. extra parenchymal	5.45	1.70–17.51 (0.004)	adjusted by laboratory (random effect)
Intra-parenchymal vs. extra parenchymal	9.99	2.11–47.21 (0.004)	adjusted by test (fixed effect) and laboratory (random effect)

**Table 10:** Number of fecal samples tested by each laboratory per assay.

Micro-Copro Ring Trial Test	Laboratory Code												
	B	C	D	E	F	H	L	R	S	W	Y	Z	Total
	N^o^ of fecal samples												
Saline and iodine wet mount preparations of fecal smears for direct examination	5	5	0	0	0	5	5	0	0	5	5	5	35
Microscopic examination after concentration (whether by sedimentation or flotation)	5	5	5	5	5	5	5	5	5	5	5	0	55
Kato-Katz followed by Ziehl-Neelsen staining [29]	0	0	0	0	0	0	0	0	0	0	0	5	5
Total	10	10	5	5	5	10	10	5	5	5	10	10	95
Molecular-Copro Ring Trial test													
rt-PCR ^1^ (Tsol 9) [30,31]	0	5	0	0	0	0	0	0	0	0	0	0	5
rt-PCR (HDP2) [32]	0	5	0	0	0	0	0	0	0	0	0	0	5
c-PCR ^2^ (Tsol 9) [30,31]	0	5	0	0	0	0	0	0	0	0	0	0	5
c-PCR (HDP2) [32]	0	5	0	0	0	0	0	0	0	0	0	0	5
PCR [33]	5	0	0	0	0	0	0	0	0	0	0	0	5
PCR [34]	5	0	0	0	0	0	0	0	0	0	0	0	5
PCR [35]	0	0	0	0	0	0	5	0	0	0	0	0	5
c-PCR (ITS)	0	0	0	0	0	0	0	0	5	0	0	0	5
PCR [30]	0	0	5	0	0	0	0	0	0	0	0	0	5
PCR-RFLP ^3^ [36]	0	0	5	0	0	0	0	0	0	0	0	0	5
Semi-nested PCR + RFLP [37]	0	0	0	5	0	0	0	0	0	0	0	0	5
Sanger sequencing	5	0	0	0	0	0	0	0	0	0	0	0	5
Total	15	20	10	5	0	0	5	0	5	0	0	0	60

^1^ real time-PCR; ^2^ conventional-PCR; ^3^ PCR-restriction fragment length polymorphism.

**Table 11:** Diagnostic accuracy by test of the microscopic copro-Ring Trial.

Test	TN ^1^	FP ^2^	FN ^3^	TP ^4^	Total Samples	Sensitivity (%)	Specificity (%)	Correctly Classified (Reliability)	ROC Area (Accuracy)	95% IC
Saline and iodine wet mount preparations of fecal smears for direct examination	14	0	0	21	35	100	100	100	1	(0.99–1.0)
Microscopic examination after concentration (whether by sedimentation or flotation)	21	1	1	32	55	96.97	95.45	96.36	0.962	(0.87–0.99)
Kato-Katz followed by Ziehl-Neelsen staining [29]	2	0	0	3	5	100	100	100	1	(0.48–1.0)

^1^ True negative; ^2^ false positive; ^3^ false negative; ^4^ true positive.

**Table 12:** Diagnostic performance by test of the molecular copro-Ring Trial.

Molecular-Test	TN ^1^	FP ^2^	FN ^3^	TP ^4^	Total Samples	Sensitivity (%)	Specificity (%)	Correctly Classified (Reliability, %)
rt-PCR ^5^ (Tsol 9) [30,31]	2	0	1	2	5	66.6	100	80
rt-PCR (HDP2) [32]	2	0	2	1	5	33.3	100	60
c- PCR ^6^ (Tsol 9) [30,31]	2	0	2	1	5	33.3	100	60
c- PCR (HDP2) [32]	2	0	2	1	5	33.3	100	60
PCR [33]	2	0	3	0	5	-	100	40
PCR 34]	2	0	2	1	5	33.3	100	60
PCR [35]	2	0	0	3	5	100	100	100
c-PCR (ITS)	2	0	3	0	5	-	100	40
PCR [30]	2	0	2	1	5	33.3	100	60
PCR-RFLP ^7^ [36]	2	0	2	1	5	33.3	100	60
Semi-nested PCR + RFLP [37]	2	0	2	1	5	33.3	100	60
Sanger sequencing	2	0	2	1	5	33.3	100	60

^1^ True negative; ^2^ false positive; ^3^ false negative; ^4^ true positive; ^5^ real time PCR; ^6^ conventional PCR; ^7^ PCR-restriction fragment length polymorphism.

## Data Availability

Data are available after request.

## Supplementary Materials

The following are available online at https://www.mdpi.com/article/10.3390/microorganisms9061173/s1, Supplementary Table S1: Breakdown of the microscopic-coprological Ring Trial results; Supplementary Table S2: Breakdown of the molecular-coprological Ring Trial results.
